# Preliminary safety and efficacy profile of prucalopride in the treatment of systemic sclerosis (SSc)-related intestinal involvement: results from the open label cross-over PROGASS study

**DOI:** 10.1186/s13075-017-1340-y

**Published:** 2017-06-20

**Authors:** Barbara Vigone, Monica Caronni, Adriana Severino, Chiara Bellocchi, Anna Rita Baldassarri, Mirella Fraquelli, Gaia Montanelli, Alessandro Santaniello, Lorenzo Beretta

**Affiliations:** 1 Scleroderma Unit, Referral Center for Systemic Autoimmune Diseases, Fondazione IRCCS Ca’ Granda Ospedale Maggiore Policlinico di Milano, Via Pace 9, 20122 Milano, Italy; 2 Gastroenterology and Endoscopy Unit, Fondazione IRCCS Ca’ Granda Ospedale Maggiore Policlinico di Milano, Via Francesco Sforza 35, Milano, Italy

**Keywords:** Systemic sclerosis, Intestinal involvement, Constipation, Serotonin agonist

## Abstract

**Background:**

Prokinetics are used to treat enteric dismotility symptoms in systemic sclerosis (SSc) patients, but they often lack adequate efficacy. The most effective prokinetics belonging to the serotonin (5-HT_4_) receptor agonists class were withdrawn due to cardiac toxicity in relation to modest 5-HT_4_ receptor affinity. Prucalopride is a high-affinity 5-HT_4_ receptor agonist with no major cardiac issues, for which the efficacy in SSc has not yet been assessed.

**Methods:**

Forty patients with self-reported mild to moderately severe enteric symptoms were enrolled in a cross-over 2 × 2 study. Subjects were randomized 1:1 to prucalopride 2 mg/day or no treatment for one month and vice versa after a 2-week washout period. Before and after each sequence the patients compiled the University of California Los Angeles gastrointestinal tract (UCLA GIT) 2.0 questionnaire and the numbers of complete intestinal movements were recorded. Oro-cecal transit time (OCTT) was evaluated by lactulose breath test in a subgroup of patients. Data were evaluated by mixed linear models corrected for the number of laxatives used during the study periods.

**Results:**

There were 29 subjects who completed the study; 7 subjects withdrew due to side-effects and 4 subjects were not compliant with the study procedures. As compared to dummy treatment, prucalopride was associated with more intestinal evacuations (*p* < 0.001), improvement of UCLA GIT constipation (-0.672 ± 0.112 vs 0.086 ± 0.115; *p* < 0.001), reflux (-0.409 ± 0.094 vs 0.01 ± 0.096; *p* < 0.005) and bloating (-0.418 ± 0.088 vs -0.084 ± 0.09; *p* = 0.01) scores. Treatment was ranked moderately to more than moderately effective by 22 patients (72.4%). OCTT was significantly reduced during prucalopruide consumption (prucalopride: -20.1 ± 20.1 vs no treatment: 45.8 ± 21.3 minutes; treatment effect = -65.9 minutes; *p* = 0.035).

**Conclusions:**

The safety profile of prucalopride in SSc is similar to what is known from the literature. In patients with mild to severe gastrointestinal problems, prucalopride may be effective in treating dismotility symptoms, increasing the number of complete bowel movements and improving bowel transit, reducing reflux disease and bloating.

**Trial registration:**

EU Clinical Trial Registry, EudraCT2012-005348-92. Registered on 19 February 2013.

## Background

Systemic sclerosis (SSc) is an autoimmune disease characterized by widespread vasculopathy, immune system activation and fibrosis of the skin and of the internal organs [1]. The gastrointestinal tract (GIT) is frequently affected in SSc with up to 90% of patients presenting with upper or lower GIT involvement [2] as a consequence of motor disturbances [3–5] secondary to myenteric neuropathy, muscle atrophy and fibrosis [6]. The reduction of enteric propulsive forces leads to symptoms of enteric dismotility syndromes, such as constipation, bacterial overgrowth with bloating, diarrhea and malabsorption and, in the most severe cases, acute or chronic pseudo-obstruction may occur [7].

The treatment of SSc-related intestinal involvement is challenging and often limited to supportive measures [8]. The use of prokinetcs has been advocated to ameliorate dismotility symptoms in SSc patients, yet often with disappointing results, especially in those with more advanced disease. Among prokinetics, the ones with the most favorable therapeutic profile are those belonging to the serotonin (5-HT_4_) receptor agonists class. As a prototypical representative of this class, cisapride has been shown to ameliorate gastric contractions and increase esophageal amplitude waves [9] and to reduce the colonic transit time in SSc patients [10]. Nonetheless, cisapride has been withdrawn from the global market due to safety concerns related to cardiac toxicity [11, 12]. These side-effects were linked to the lack of selectivity for the for the 5-HT_4_ receptor and to the blockade of the human ether-a-go-go-related gene (hERG)-encoded K^+^ channel. The consequences of this interaction are the prolongation of cardiac action potential repolarisation and, thus, QT interval, leading to a clinically significant arrhythmogenic potential [12, 13]. On the contrary, novel agonists that specifically interact with the 5-HT_4_ receptor and possess GIT tissue-specific agonist activity, have a favorable cardiac profile and no major cardiac issues. Among those, prucalopride, a member of the benzofurancarboxamide agonists possesses markedly increased selectivity for the 5-HT_4_ receptor as compared to cisapride (150 times vs <1, respectively) [14]. In vitro, high concentrations of prucalopride are equivalent to 5-HT in stimulating peristalsis [14] and in vivo, prucalopride dose-dependently stimulates high-amplitude clustered contractions in the proximal colon and inhibits contractile activity in the distal colon [15]. In humans, prucalopride has proved effective in treating chronic constipation unresponsive to laxatives, improving stool frequency, stool consistency, straining and quality of life [16–18]. Results of mechanistic studies in patients with idiopathic constipation suggest the usefulness of prucalopride in patients with an associated upper or generalized gastrointestinal motility disorder [18]. Prucalopride does not interact with hERG channels and in large placebo-controlled studies no differences in vital signs or electrocardiogram parameters have been observed between groups [16, 17, 19].

In relation to its safety and efficacy profile, the use of prucalopride has been postulated in SSc [2]; however, only one report describing two cases of SSc patients with GIT unresponsive to other prokinetics has been published so far [20]. To increase our knowledge about prucalopride and to evaluate its therapeutic potential in SSc, we conducted a randomized crossover trial in subjects with mild to severe constipation, providing evidence for a beneficial effect on enteric symptoms and health-related quality of life (HQoL).

## Methods

### Patients and study procedures

A cross-over 2 × 2 randomized controlled trial comparing prucalopride to no treatment was designed. Forty female subjects with SSc and chronic constipation were randomized 1:1 to receive prucalopride 2 mg/day or no treatment for one month and vice versa after a 2-week washout period, according to the ABBA design. Recruitment was limited to female subjects as prucalopride is not currently approved for the treatment of chronic constipation in men.

All the subjects fulfilled the American College of Rheumatology (ACR)/European League Against Rheumatism (EULAR) 2013 criteria for the classification of SSc [21], the Rome III criteria for functional constipation (Table 1a) [22] and had mild-to-severe subjective symptoms of constipation on a 5-point Likert scale (Table 1b) (minimum and maximum allowed score = 1 and 3, respectively) [23].

**Table 1:** Scales and criteria used for patient selection

a. Rome III diagnostic criteria for functional constipation [22]	
1. Must include* two or more of the following: - Straining during at least 25% of defecations - Lumpy or hard stools in at least 25% of defecations - Sensation of incomplete evacuation for at least 25% of defecations - Sensation of anorectal obstruction/blockage for at least 25% of defecations - Manual manoeuvres to facilitate at least 25% of defecations (e.g., digital evacuation, support of the pelvic floor) - Fewer than three defecations per week 2. Loose stools are rarely present without the use of laxatives 3. Insufficient criteria for irritable bowel syndrome *criteria fulfilled for the last 3 months.	
b. Likert scale for the subjective evaluation of constipation [23]	
0. Absent 1. Mild 2. Moderate 3. Severe 4. Very severe	

The number of complete spontaneous bowel movements (CSBMs) per month (4 weeks) and the proportion of patients reporting ≥3 defecations per week were used as study endpoints according to the European Medicines Agency (EMA) recommendations [24] and to previous registration trials [16, 17]. To this end, patients were given a diary and were instructed to record their daily CSBMs throughout the study periods [16, 17]. The use of bisacodyl 10 mg as laxative was allowed and the number of used tablets was recorded as well. Patients were not allowed to take any other laxatives or to perform enemas to treat constipation.

Additionally, before and after each treatment period the patients compiled the Italian version of the UCLA GIT 2.0 questionnaire [25], the 5-point Likert scale for constipation (Table 1a) and a modified 4-point Likert scale for gastroesophageal symptoms (0: no symptoms, no burning sensation; 1: mild symptoms, self-awareness of burning but well-tolerated; 2: moderate symptoms, initially incapacitating burning sensation that may impair normal activities, including rest; 3: severe symptoms, incapacitating burning sensation that impairs normal activities, including rest).

Finally, the patients globally evaluated the efficacy of treatment using a 5-point scale [26] (0: not at all effective; 1: a little effective; 2: moderately effective; 3: quite effective; 4: extremely effective).

None of the selected patients made use of laxatives the week before the initiation of the study procedures or was treated with systemic antibiotics the preceding month.

### Explorative analysis of the OCTT

To evaluate the effect of prucalopride on the OCTT, patients were also given the possibility to perform a lactulose breath test (LBT) [27] at the beginning and at the end of each study period. The procedure was performed in accordance with accepted criteria [28] in fasting conditions, measuring H_2_ breath concentration by Gastro + ™ Gastrolyzer® (Bedfont Scientific Ltd, Station Road, Harrietsham, Maidstone, Kent, ME17 1JA, UK) in basal conditions and every 10 minutes for at least 3 h after the administration of an oral loading dose of lactulose (10 g in 100 ml of water). The OCTT results were interpreted as previously described [28].

### Statistical analysis

To compare treatment effects and to account for carry-over effects, mixed linear models were used, correcting the results for the number of laxatives used during the study periods [29]. Estimated marginal means corrected for the number of laxatives ± estimated standard errors (SE) are presented. For categorical variables, binary logistic multivariate models were used. To test the correct allocation into the study arms, the paired samples *t* test and the Fisher exact test were performed. Results of descriptive statistics, binary logistic regression, the *t* test and the Fisher test are presented as mean ± standard deviation (SD). Statistical analyses were performed using the SPSS 23.0 software (IBM Corp, Armonk NY, USA).

The sample size for the cross-over trial was determined based on a theoretical 66.6% increase in CSBMs in the treatment arm vs the non-treatment arm (20 vs 12 CSBMs/month) with a within-patients SD equal to 12 CSMBs. With these parameters, 40 patients were required to reject the null hypothesis of non-superiority (one-sided 0.05 alpha) with power = 0.9 (n = 30 required for power = 0.8). Calculations were performed using the sample size online calculator provided by the Massachusetts General Hospital's Biostatistics Center (http:// http://hedwig.mgh.harvard.edu/biostatistics/).

## Results

**Fig. 1:**
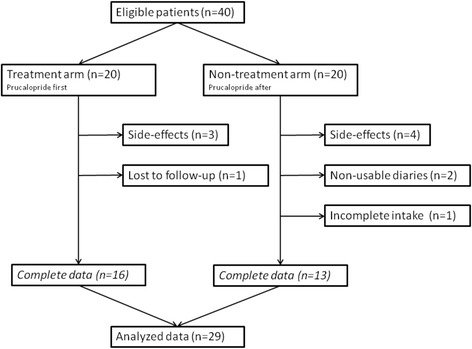
Flow chart of patients included in the study

Overall 29 participants completed all the study procedures (Fig. 1). Seven patients (17.5%) did not tolerate prucalopride due to side effects; the reported adverse events were: headache (three subjects), abdominal pain (two subjects), dizziness (one subject) and the sensation of feeling sick (one subject). In most cases (four out of seven) side effects were observed the first day of treatment and were considered severe enough to preclude the prosecution of the study, despite the proposal of mitigation strategies (i.e. use of pain killers); in the remaining cases attempts were made to go on with the treatment; however, this was discontinued within 1 week due to the persistence of side effects. Patients experiencing side effects were not clinically different from those who completed the study procedures in terms of disease duration, autoantibody profile or baseline self-reported measures of GIT disease severity (results not shown). Four patients were finally excluded from the analysis (10%) because not they were not compliant with the study procedures (badly or non-compiled diaries and self-efficacy forms including those completed during the non-treatment period, *n* = 2), because of inadequate drug intake (*n* = 1) or because the patient did not attend the assessment visit (*n* = 1).

The clinical and baseline characteristics of the remaining 29 study participants are reported in Table 2. Randomization was well-balanced with no statistical differences between arms, except for older age in participants who took the study drug first. Overall, 10 patients (34.5%) rated their constipation as “severe”, 17 (58.6%) as “moderate” and 2 (6.9%) as “mild”.

**Table 2:** Demographics and baseline clinical characteristics

Variable	Patients with SSc (*n* = 29)	Arm 1 (drug first, *n* = 16)	Arm 2 (drug after, *n* = 13)
Age, years	54.4 ± 10.5	59 ± 9.2	48.8 ± 9.5*
Disease duration, years	12.3 ± 8.7	13.7 ± 9.9	10.7 ± 7.1
lcSSc, *n* (%)	22 (75.8)	12 (75%)	10 (79.6)
Autoantibody, *n* (%)			
ANA ACA Topo I	27 (93.1) 18 (62.1) 9 (31)	15 (93.7) 11 (68.8) 4 (25)	12 (92.7) 7 (53.8) 5 (38.5)
Likert GERD (0–4)	2.24 ± 0.74	2.25 ± 0.68	2.23 ± 0.83
Likert constipation (0–4)	2.24 ± 0.64	2.44 ± 0.63	2 ± 0.58
UCLA GIT 2.0	0.99 ± 1.,28	1.23 ± 1.65	0.69 ± 0.49
UCLA GIT 2.0 constipation	1.27 ± 0.67	1.31 ± 0.68	1.21 ± 0.68
UCLA GIT 2.0 subscales			
Reflux Bloating Fecal soilage Diarrhea Social activities Emotional wellbeing	1.01 ± 0.69 1.48 ± 0.9 0.51 ± 0.91 0.22 ± 0.41 0.72 ± 0.59 0.77 ± 0.78	1 ± 0.76 1.5 ± 0.97 0.61 ± 1.02 0.34 ± 0.47 0.83 ± 0.68 0.86 ± 0.85	1.02 ± 0.63 1.46 ± 0.85 0.38 ± 0.77 0.08 ± 0.28 0.59 ± 0.44 0.67 ± 0.71

Values expressed as mean ± standard deviation except where otherwise indicated. *SSc* systemic sclerosis, *lcSSc* limited cutanoeus SSc, *ANA* antinuclear antibodies, *ACA* anticentromere antibodies, *Topo I* anti-Topomerase I antibodies, *GERD* gastroesophageal reflux disease, *UCLA GIT* University of California Los Angeles gastrointestinal tract questionnaire

*p < 0.05 vs Arm 1

### Prucalopride improves the number of CSBMs and ameliorates constipation in patients with SSc

**Fig. 2:**
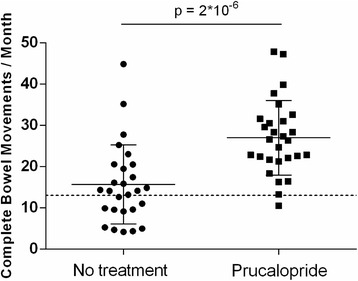
Effect of prucalopride on defecation. Number of complete spontaneous bowel movements recorded by patients during the 1-month consumption of prucalopride 2 mg/day or the 1-month period without treatment. The plot represents the values adjusted for the mean of used laxatives. *Dashed lines* indicate the occurrence of ≥3 evacuations/week (12/month)

During the treatment period, patients achieved more complete bowel movements than during the non-treatment period (Fig. 2). The number of patients reporting ≥3 defecations per week was 25 (86.2%) and 14 (48.3%) in the two arms, respectively; these results were significant in binary logistic models that accounted for the use of laxatives and the randomization order (*p* = 0.014). In this model, patients receiving prucalopride were five times more likely to have ≥3 defecations per week than when they did not receive the study drug (odds ratio = 5.2, 95% confidence interval 1.39–19.2).

**Fig. 3:**
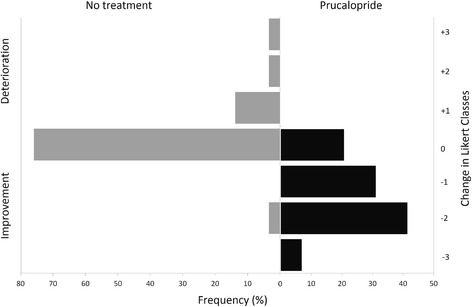
Change in Likert scale for the evaluation of constipation. Change in the grading of the Likert scale for constipation at the end of the study periods. Negative values (*black bars*) indicate improvement; positive values (*gray bars*) indicate deterioration. Improvement was almost exclusively observed in patients in the active treatment arm

Treatment with prucalopride was associated with the improvement of constipation symptoms and of gastroesophageal reflux disease (GERD) as assessed both by Likert scales and by the specific UCLA GIT 2.0 subscales (Table 3); bloating was also significantly reduced by the treatment. The treatment effect on constipation could be rated as “much better” according to the minimally important differences in the UCLA GIT 2.0 questionnaire reported in [30]; the treatment effect on GERD and bloating could be rated as “somewhat better” (Table 3). Out of the 29 patients, 20 (69%) had changes in the UCLA GIT 2.0 constipation scores greater than the lower bound of the 95% confidence interval for marked responses [30]. Accordingly, prucalopride was ranked as moderately to extremely effective by 22 patients (72.4%) (extremely effective = 34.5%; quite effective = 37.9%; moderately effective = 20.7%; little effective = 6.9%). A reduction in the subjective grading of constipation was observed in patients in the active treatment arm, while at the end of the no-treatment period they mostly reported no change in the severity of constipation (Fig. 3).

**Table 3:** Effect of treatment

Variable	Change with prucalopride^a^	Change without treatment^a^	Effect size^a^	Rating^b^	*p*
Likert GERD	-0.678 ± 0.108	-0.001 ± 0.114	-0.677	NA	7.8*10^-5^
Likert constipation	-1.282 ± 0.155	0.135 ± 0.158	-1.417	NA	2.01*10^-7^
UCLA GIT 2.0	-0.147 ± 0.061	0.021 ± 0.063	-0.168	Same	0.047
UCLA GIT 2.0 Constipation	-0.672 ± 0.112	0.086 ± 0.115	-0.758	Much better	5.4*10^-5^
UCLA GIT 2.0 Subscales					
Reflux Bloating Fecal soilage Diarrhea Social activities Emotional wellbeing	-0.409 ± 0.094 -0.418 ± 0.088 -0.097 ± 0.133 0.367 ± 0.093 -0.106 ± 0.11 -0.214 ± 0.093	0.01 ± 0.096 -0.084 ± 0.09 0.074 ± 0.141 0.089 ± 0.097 -0.006 ± 0.112 0.017 ± 0.093	-0.419 -0.334 -0.171 0.277 -0.1 -0.231	Better Better Same Worse Better Same	0.003 0.011 0.38 0.053 0.503 0.051

Change in specific scales and scores used to evaluate gastro-intestinal involvement in the period after treatment (prucalopride) or in the no-treatment 4-week periods. Values presented as estimated marginal mean ± estimated standard error corrected for the number of laxatives. *GERD* gastroesophageal reflux disease, *UCLA GIT* University of California Los Angeles gastrointestinal tract questionnaire, *NA* not available. ^a^Negative values indicate improvement. ^b^Interpretation of treatment effect size according to [30]

### OCTT is favorably influenced by prucalopride and correlates with the number of CSBMs

Sixteen patients participated in the LBT substudy and had complete LBT evaluations before and after the treatment/no treatment study periods. Baseline OCTT was 127 ± 70 minutes. One-month treatment with prucalopride led to a reduction in the OCTT that, on the contrary, increased in the no-treatment arm. Allowing for the order of treatment administration and the concurrent therapy with bisacodyl in the model, these differences were statistically significant in favor of the study drug (prucalopride: -20.1 ± 20.1 vs no-treatment: 45.8 ± 21.3 minutes; treatment effect = -65.9 minutes; *p* = 0.035). The change in the OCTT was an independent predictor of the number of complete bowel movements in a mixed model that accounted for the carry-over effect of the treatment and for the concurrent therapy with laxatives (*p* = 0.011) (direction of correlation: reduction in the OCTT, increase in the number of defecations). Similarly, changes in the perceived severity of constipation according to Likert scales directly correlated with changes in the OCTT (*p* = 0.014), yet there was no correlation with change in the UCLA GIT 2.0 constipation scores.

## Discussion

The use of prokinetics has been advocated to treat enteric dismotility symptoms in SSc [8]; however, evidence in favor of this approach is lacking. In the EULAR recommendations for the treatment of SSc, the strength of the recommendation in favor of prokinetics is ranked as “C” due to the lack of proper controlled trials. The major source of evidence comes from studies on upper GIT disturbances treated with cisapride [8, 9], and there is just one report that this unselective 5-HT_4_ receptor agonist may accelerate colonic transit in a small unselected case series of patients with SSc [10]. Safety issues related to this class of drugs that led to their marked withdrawal may have frustrated further attempts to study the potential of prokinetics in SSc-related enteric dismotility symptoms. However, it comes as a surprise that newer agents with no major cardiac issues have not been evaluated so far [12–14].

Our study clearly illustrates the beneficial effect of prucalopride in selected SSc patients with symptoms of constipation. In our work, we focused on patients with mild-to-severe colonic functional alterations, while we excluded patients with self-reported very severe dismotility symptoms. Indeed, in this subset of patients, prokinetics are likely to be ineffective due to end-stage fibrosis and the ablation of myenteric propulsive forces [6]. Despite these restrictions, constipation was quite severe in patients according to the average UCLA GIT 2.0 constipation scores [31] and to Likert scales, and considering the relevant number of cases (>50%) with fewer than three complete spontaneous bowel movements per week. All the aforementioned parameters used to evaluate the severity of constipation were positively affected by prucalopride. The UCLA GIT 2.0 constipation scores were markedly reduced after treatment, with an effect size that would classify the drug response as “much better” according to the minimally important differences of the UCLA GIT questionnaire [30]. Accordingly, about 70% of study participants ranked the efficacy of the treatment as very/extremely effective. Finally, the proportion of patients achieving ≥3 defecations per week, which is the threshold to define normal colonic function [21], was much higher in the treatment arm than in the non-treatment arm.

The improvement in colonic function that is observed after treatment with prucalopride is partially attributable to accelerated colonic transit times, as shown by the reduction in the OCTT in the LBT. These results are in accordance with a previous prucalopride study conducted in subjects with chronic constipation [32] according to the Rome III criteria [21], which we also used to select our patients. The correlation between the change in the subjective severity of constipation as measured by Likert scale (i.e. its improvement) or the increased number of defecations and an accelerated OCTT is also in accordance with the aforementioned work [32]. Of interest, in this report patients receiving placebo had increased OCTT and also in another report [33] dummy treatment was associated with a worsening of transit time. Therefore, the increase in OCTT we observed in the no-treatment arm is not surprising; however, we advise some caution in the interpretation of the results as the increase in OCTT in the no-treatment arm was a major contributor to the significance of results. Overall, considering the limited number of participants that completed this additional study procedure, OCTT results should be carefully gauged and considered mostly explorative.

Treatment with prucalopride was not only associated with an improvement in intestinal symptoms, including bloating, but also with a reduction in the subjective severity of GERD as assessed by the dedicated Likert scale and by the specific UCLA GIT 2.0 subscale. In a previous study prucalopride was shown to have no effect on the lower esophageal sphincter, esophageal motility or total reflux events; however, it was capable of improving acid clearance time and of increasing gastric emptying [34] contributing thus to the amelioration of GERD symptoms. These results are quite relevant considering that on average the severity of reflux in our population could be graded as severe to very severe in relation to the reflux UCLA GIT 2.0 subscale [31].

Despite its efficacy, treatment with prucalopride is characterized by a number of adverse events that precluded the prosecution of the treatment in 17.5% of patients. These figures are slightly worse, but substantially in accordance with previous phase III trials showing that discontinuation rates may be as high as 15% [17]. In relation to the study design, we cannot rule out the possibility that the large number of side effects we observed might somewhat be due to the open-label administration of the study drug. As in previous reports, in our study, many discontinuations were the consequence of adverse events that occurred on the first day of drug administration. Besides that, the increase in intestinal propulsive forces may promote defecation to such an extent that it may be perceived as diarrhoea, as indicated by the worsening of the specific UCLA GIT 2.0 subscale.

The major limitation of our study is the lack of a placebo arm, as patient-reported outcomes that constitute the core of our evaluation are largely subjective. This problem is partially addressed, yet incompletely circumvented by the OCTT substudy demonstrating the functional efficacy of prucalopride. Thus, the results of our research should be considered preliminary, yet our encouraging findings provide evidence and support for larger, controlled studies. Finally, our study does not address the question about the long-term efficacy of prucalopride and dose escalation to reduce side-effects and increase adherence to treatment.

## Conclusions

Despite limitations linked to the nature of the study, prucalopride seems to be well-tolerated and effective in patients with SSc with mild to moderately severe constipation, favoring defecation, reducing bloating and ameliorating GERD. A careful evaluation of patients may be necessary to select those that may most benefit from this kind of treatment. Further larger and controlled studies may thus be warranted to address this issue and to assess the efficacy of prucalopride in a wider population of patients with scleroderma.

## Abbreviations

5-HT_4_: Serotonin
CSBMs: Complete spontaneous bowel movements
EULAR: European League Against Rheumatism
GERD: Gastroesophageal reflux disease
GIT: Gastrointestinal tract
LBT: Lactulose breath test
OCTT: Oro-cecal transit time
SD: Standard deviation
SSc: Systemic sclerosis
